# Stability Indicating High-Performance Liquid Chromatography Method for the Estimation of Artemether in Capsule Dosage Forms

**DOI:** 10.4103/0975-1483.62220

**Published:** 2010

**Authors:** A Shrivastava, R Issarani, BP Nagori

**Affiliations:** *Department of Pharm. Sciences, B.R. Nahata College of Pharmacy, Mhow-Neemuch Road, Mandsaur (M.P) - 458 001, India*; 1 *L.M. College of Science and Technology, Pharmacy wing, Shastri Nagar, Sector A, Jodhpur (Rajasthan), India*

**Keywords:** ART, forced degradation, high-performance liquid chromatography, stability indicating method of artemether

## Abstract

A new simple, sensitive, precise, and accurate high-performance liquid chromatography (HPLC) method of analysis for artemether both as a bulk drug and in capsule formulations was developed and validated. The method employed mobile phase acetonitrile (ACN) and buffer in the ratio 65:35 of pH 6.5 adjusted with tryethylamine. The linear regression analysis data for the calibration plots showed good linear relationship with *r*^2^ = 0.9996 in the concentration range 250-750 μg/ml. The mean value slope and intercept were 9355.5 and −93.5, respectively. The method was validated for precision, accuracy, and recovery studies. Limit of detection (LOD) and Limit of quantitation (LOQ) for artemether were found to be 21.83-750 μg/ml, respectively. The method has been successfully applied in the analysis of marketed capsule formulations. The presented method was found to be reliable to separate all the degradents from all the stress conditions with resolution of more than 1.5 showing that it is a stability indicating method.

## INTRODUCTION

Artemether ((3*R*, 5*aS*, 6*R*, 8*aS*, 9*R*, 10*S*, 12*R*, 12*aR*)-decahydro-10-methoxy-3, 6, 9-trimethyl-3, 12-epoxy-12*H*pyrano [4,3-j]-1, 2-benzodioxepin) is a semisynthetic polyoxygenated amorphene-containing aperoxide bridge that confers potent antimalarial activity.[1] It is the *O*-methyl ether prodrug of dihydroartemisinin and a derivative of artemisinin (qinghaosu), the principal antimalarial constituent of the Chinese herb *Artemisia annua* (qing hao).[2] Artemether is active against the erythrocytic stage of multidrug-resistant strains of *Plasmodium falciparum*. The antimalarial activity has been attributed to chemical activation of the drug within the food vacuole of the intraerythrocytic stage of the parasite; it is proposed that reductive cleavage of the peroxide bridge by heme liberated during digestion of hemoglobin generates free radicals, which induce oxidative stress and alkylate heme and vital parasite proteins.[3] An interaction with membrane phospholipids has also been suggested.[4] The peroxide group in these compounds appears essential for activity and the peroxide group is retained in the active metabolite, dihydroartemisinin.[5]

Because of the promising activity exhibited by artemether against multidrug-resistant strains of *P. falciparum*, several researchers have focused on the development of various analytical methods to determine artemether in different matrices, such as plant extracts, serum, and pharmaceutical formulations. These methods include gas UV spectrophotometry,[5,6] high-performance liquid chromatography (HPLC) based on UV absorption,[7–9] chromatography-mass spectrometry (GC-MS),[10] chemilu minescence and electrochemical detection,[11] high- performance thin-layer chromatography (HPTLC),[12,13] and the capillary electrophoresis techniques.[14] However, to the best of our knowledge no stability indicating HPLC method has been published in the available literature.

The aim of the present work is to develop and validate[15] an accurate, specific, and precise stability indicating HPLC method for determination of artemether as bulk drug and in solid dosage forms.

## MATERIALS AND METHODS

HPLC Apparatus (shimadzu) equipped with LC-10 ATvp, double reciprocating plunger pump and SPD-10 Avp UV detector, and deuterium lamp as a light source ranging 190-600 nm was used. Hypersil Octadecyl silane (ODS) column, (250 × 4) mm, 5 μm particle size of packing, pore size 120 A, Thermo Electron Corporation. Marketed formulations Larither and Falcidol capsules were purchased from a local market. Mobile phase selected was ACN and buffer in the ratio of 65:35 of pH 6.5 adjusted with tryethylamine. Wavelength selected was 210 nm. Standards and facilities were provided by Oasis Lab, Ahmedabad.

### Preparation of a mobile phase

650 volumes of acetonitrile (ACN) was mixed with 0.005 M potassium dihydrogen phosphate (KH_2_PO_4_), adjusted to pH 6.5 with triethylamine filtered and degassed.

### Preparation of 0.005 M KH_2_PO_4_

0.340 g KH_2_PO_4_ was transferred to a 500 ml volumetric flask and dissolved up to the mark with double distilled water.

### Calibration curve

Suitable aliquots of the standard stock solution (10 mg/ ml) of ART (0.25, 0.35, 0.4, 0.45, 0.5, 0.55, 0.6, 0.65 and 0.75) were taken in 10 ml volumetric flasks and diluted with a mobile phase up to the mark to get 250, 350, 400, 450, 500, 550, 600, 650, and 750 μg/ml solution of drug. The prepared solutions were then filtered through 0.45 μ filter, and 20 μl of each solution was injected.

The calibration data, the linear regression equation, and correlation coefficient for artemether were found to be *y* = 9355.5*x* − 93.7 and *r* = 0.9998, where *y* is response and *x* is the concentration of drug solution, respectively.

## VALIDATION OF PROPOSED METHOD

### Linearity

Linearity was assessed by two methods.

#### Visual examination

The numbers of points equally distributed on both sides of best fit line were inspected. From the calibration curve it is clearly seen that out of 9 points, 4 are on positive side (upper side of best fit line), 4 are on negative side (lower side of best fit line) and 1 is on the best fit line.

#### Residual analysis

Residuals were calculated and a graph was plotted between the residuals/deviations (predicted response-experimental response) and concentration. Distribution of residuals between upper and lower side of the regression line shows linearity.

### Range

#### Working range

It begins from limit of quantification to the maximum concentration used for the development of the analytical method. In this case it is equal to 21.8317-750 μg/ml.

#### Linearity range

It is the interval in which the response is directly proportional to the concentration between the upper and lower levels (which is generally ±5% of the intercept of average value). In this case it is equal to 250-750 μg/ml.

#### Target concentration

It is defined as the concentration which is equal to the midpoint of linearity range [(250 + 750)/2] = 500 μg/ml.

#### Target range

It is that concentration which is 80%, 100% and 120% of the target concentration. In this case these are equal to 400, 500, and 600 μg/ml.

### Precision

#### Repeatability

Assessed by the area of six replicate determinations of ART solution at 100% of the target concentration, i.e. at 500 μg/ml. RSD obtained because of variation in area was 0.257 showing that the method it repeatable.

#### Intraday and Interday

Method was repeated at three concentration levels, i.e. 80%, 100%, and 120% for three times in a day for intraday and in three consecutive days for interday precision. RSD values obtained were 0.643 and 0.712 for intraday and interday, respectively.

### Accuracy

5 ml of a preanalyzed capsule powder solution (500 μg/ ml) and 0.1, 0.2 and 0.3 ml of an ART standard solution (1 mg/ ml) were mixed within three different 10 ml volumetric flask, respectively. The estimation of drug was done by a proposed method. The results of recovery of Larither and Falcidol capsules are shown in Tables 1 and 2, respectively.

**Table 1:** Results of recovery of larither capsules

Conc. (μg/ml)	Conc. found before spiking (μg/ml) (C_1_)	Conc. of std added (μg/ml) (C_2_)	Conc. found after spiking (μg/ml) (C_3_)	%Recovery (C_3_–C_1_)* 100/C_2_	Mean ± S.D	RSD
250	247.558	100	345.89	99.52	99.37 ± 0.76	0.765
			344.82	99.21		
			348.67	100.32		
250	247.558	200	448.29	100.16		
			446.15	99.68		
			447.05	99.88		
250	247.558	300	539.06	98.44		
			536.93	98.06		
			542.27	99.03		

**Table 2:** Result of recovery study of falcidol caps

Conc. (μg/ml)	Conc. found before spiking (μg/ml) (C_1_)	Conc. of std added (μg/ml) (C_2_)	Conc. found after spiking (μg/ml) (C_3_)	%Recovery (C_3_–C_1_)* 100/C_2_	Mean ± S.D	RSD
250	244.75	100	340.95	98.89	99.31 ± 0.514	0.517
			342.55	99.36		
			343.6	99.66		
250	244.75	200	437.68	98.41		
			440.89	99.13		
			440.04	98.94		
250	244.75	300	545.11	100.06		
			542.68	99.62		
			543.21	99.71		

### System suitability

Five injections of the same solution (500 μg/ml) were injected and variations between area and RT were observed. RSD due to changes in area and RT were found to be 0.274 and 0.326, respectively. Asymmetric and tailing factors were 1.20 and 1.30, respectively. The number of theoretical plates for the test concentration was 6998.

### Limit of detection and limit of quantitation

The value of limit of detection (LOD) and limit of quantitation (LOQ) were calculated from the formula 3.3 × (SD/S) and 10 × (SD/S), respectively, by using mean SD of data from the calibration curve. In this proposed method, 7.204 and 21.8317 μg/ml of ART are calculated as LOD and LOQ, respectively.

### Estimation of artemether in a capsule dosage form

50 mg equivalent powder of a capsule dosage form was transferred to four different 50 ml volumetric flasks. 65 ml of ACN was added and sonicated for 15 min. The flasks were then diluted up to a volume with 0.005 M phosphate buffer, filtered with 0.45 μm filter, and 20 μl was injected.

A standard artemether solution was prepared by dissolving 100 mg of drug in a 10 ml volumetric flask and diluting up to the volume. 0.5 ml of this solution was transferred to a 10 ml volumetric flask. Results of estimation of drug in two different capsule formulation are given in Table 3.

**Table 3:** Estimation of artemether in capsule dosage form

Brand name	Label claim (mg)	Conc. found (mg)	Mean ± SD	%Drug found	Mean% ± SD	%RSD
A	40	39.678	39.734 ± 0.1304	99.33	99.37 ± 0.31324	0.315
		39.904		99.76		
		39.598		98.99		
		39.756		99.39		
B	40	39.444	39.388 ± 0.13179	98.61	98.47 ± 0.32946	0.334
		39.542		98.85		
		39.323		98.31		
		39.243		98.11		

Brand A - Larither caps; Brand B - Falcidol caps

The calculation was done by using the formula:

$$\mathrm{Artemether}\;\mathrm{content}\;\left(\mathrm{mg}\right)\;=\;\left(\mathrm{Area}\;\mathrm{test}/\mathrm{Area}\;\mathrm{std}\right)\;\times \;\left(100\times 10\right)\;\times \;\left(0.5\times 10\right)\;\times \;\left(50/\mathrm{wt}\;\mathrm{capsule}\;\mathrm{powder}\right)\;\times \;\left(\mathrm{potency}\;\mathrm{of}\;\mathrm{std}/100\right)\;\times \;\left(\mathrm{average}\;\mathrm{wt}\;\mathrm{powder}/\mathrm{cap}\right)$$

### Forced degradation studies

#### Acid hydrolysis

**Figure 1:**
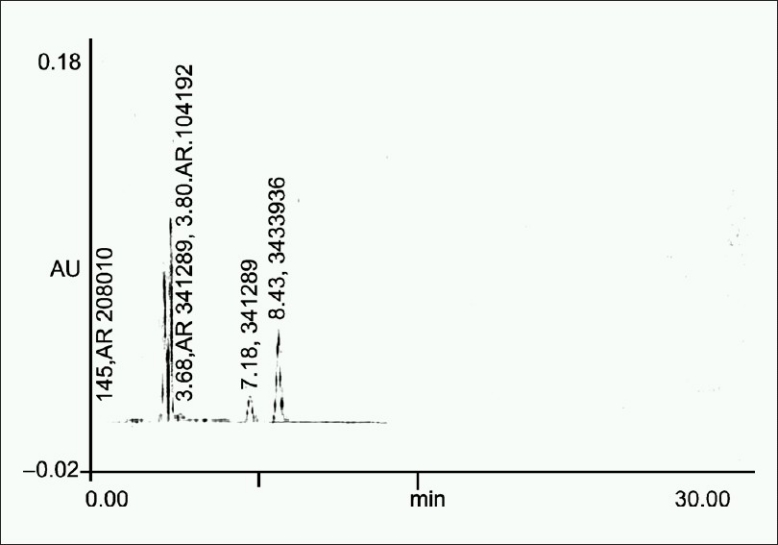
Chromatogram obtained after treating a 0.5 ml of the standard stock solution with 1 ml of 0.01 N HCl at 60°C for 3 h

0.5 ml of a standard stock solution (10 mg/ml) was transferred to a 10 ml volumetric flask and in this 1 ml of 0.01 N HCl, volume was made to about 5 ml with mobile phase and heated on the water bath at 60°C for 3 hours. Flask was then cooled to room temperature and diluted up to mark with mobile phase. Solution was then filtered through 0.45 μm filter, and 20 μl solution was injected [Figure 1].

#### Alkaline hydrolysis

**Figure 2:**
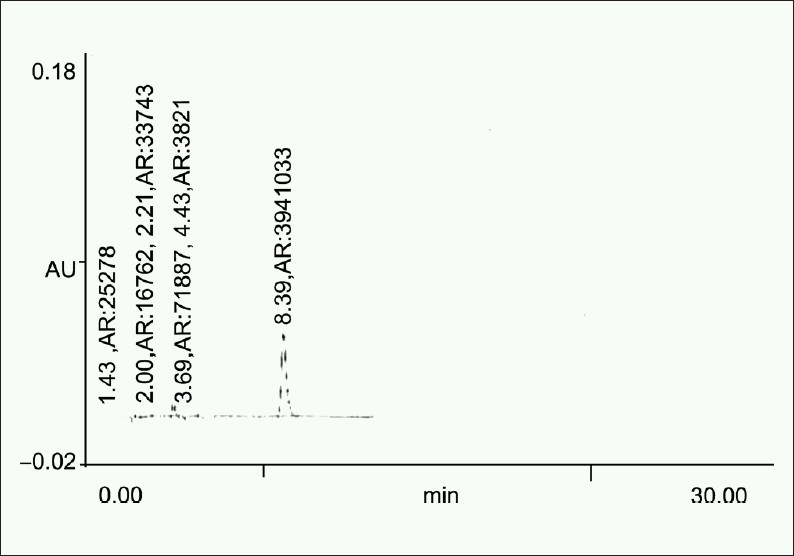
Chromatogram obtained after treating a 0.5 ml of the standard stock solution with 1 ml of 0.001 N NaOH at 60°C for 6 h

0.5 ml of standard stock solution (10 mg/ml) was transferred to 10 ml volumetric flask and in this 1 ml of 0.001 N NaOH, volume was made to about 5 ml with mobile phase and heated on the water bath at 60°C for 6 hours. Flask was then cooled to room temperature and diluted up to mark with mobile phase. Solution was then filtered through 0.45 μm filter and 20 μl solution was injected [Figure 2].

#### Oxidation

**Figure 3:**
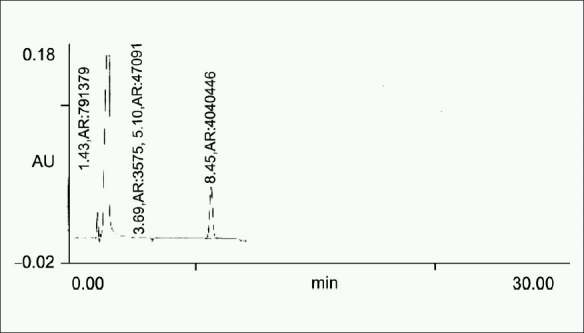
Chromatogram obtained after treating a 0.5 ml of the standard stock solution with 3% H_2_O_2_ kept overnight at RT for 24 h

0.5 ml of the stock solution (10 mg/ml) was taken in a 10 ml volumetric flask and 1 ml of 3% H_2_O_2_ was then added. The solution was then kept at room temperature for 18 h. The solution was then filtered through 0.45 μm filter, and 20 μl solution was injected [Figure 3].

### Thermal degradation

#### Dry heat

**Figure 4:**
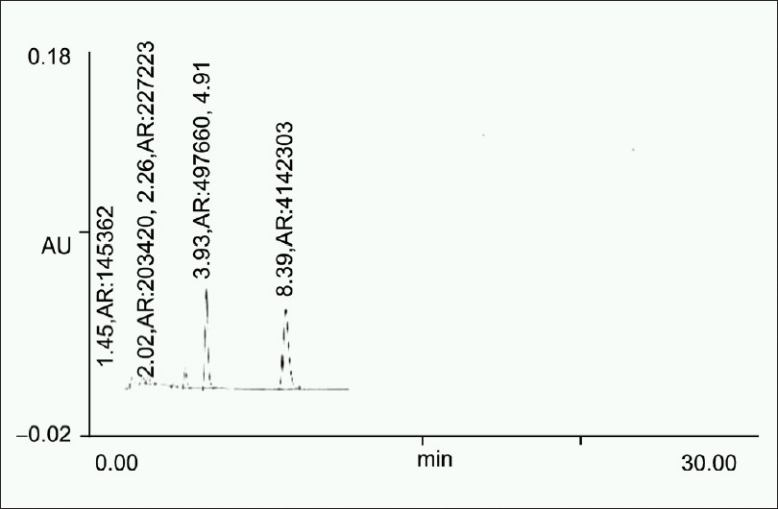
Chromatogram obtained after heating drug at 60°C for 24 h

100 mg drug was taken in a 10 ml volumetric flask and heated in the oven at 60°C for 24 h, diluted up to the mark with the mobile phase. The solution was then filtered through 0.45 μm filter, and 20 μl solution was injected [Figure 4].

#### Wet heat

**Figure 5:**
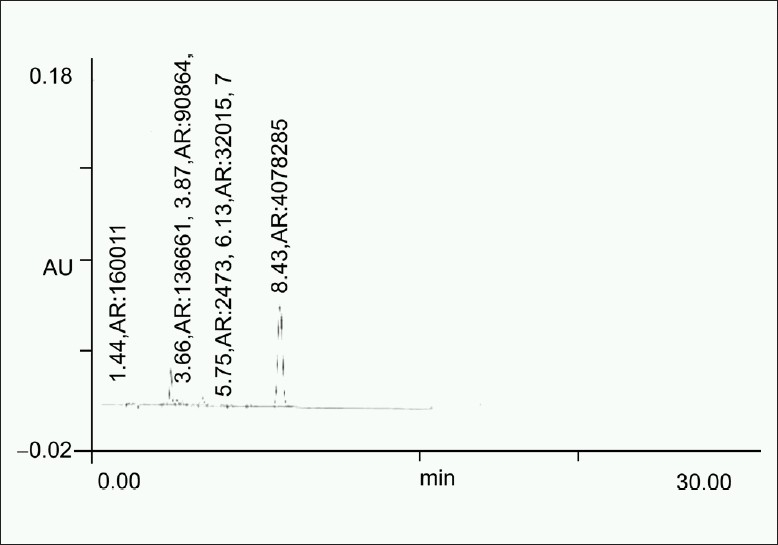
Chromatogram obtained after heating a 0.5 ml of stock solution diluted to about 5 ml heated on the water bath at 100°C for 4 h

0.5 ml of the standard stock solution (10 mg/ml) of drug was transferred to a 10 ml volumetric flask and diluted with about 5 ml of the mobile phase and kept on water bath at 100°C for 4 h. The solution was then filtered through 0.45 μm filter, and 20 μl solution was injected [Figure 5].

#### UV treatment

0.5 ml of the standard stock solution (10 mg/ml) was transferred to a 10 ml volumetric flask, diluted to the mark, and kept in UV chamber overnight for 24 h. The solution was then filtered through 0.45 μm filter, and 20 μl solution was injected, no degradation was observed.

## CONCLUSION

The developed HPLC technique is precise, accurate, specific, and stability indicating. Statistical analysis proves that the method is repeatable and selective for the analysis of artemether as bulk drug and in pharmaceutical formulations. The summary of results of stability studies and validation parameters of method are presented in Tables 4 and 5 respectively. The method can be used to determine the purity of the drug available from various sources by detecting the related impurities. It may be extended to study the degradation kinetics of artemether and for its estimation in plasma and other body fluids. As the method separates the drug from potential degradation products in different storage conditions with the resolution of more than 1.5, it is stability indicating.

**Table 4:** Summary of results of forced degradation studies

Sample exposure condition	No. of degradation products (RT)	Figure no.	%Degradation (±SD)	S.E.M	%Recovery
Acid hydrolysis	5 (3.16, 3.42, 3.68, 3.89, 7.18)	1. a	22.312 (±4.3)	0.91	77.69
Alkaline hydrolysis	5 (2.0, 2.21, 3.39, 3.69, 4.43)	1. b	15.747 (±3.8)	1.6	84.25
Oxidation	3 (2.04, 3.69, 5.10)	1. c	13.622 (± 4.2)	0.88	86.38
Heat					
Dry	5 (2.02, 2.26, 3.41, 3.93, 4.91)	1. d	11.444 (±3.6)	1.2	88.56
Wet	7 (3.66, 3.87, 4.40, 4.83, 5.75, 6.13, 7.01)	1. e	12.813 (±5.4)	2.4	87.18

**Table 5:** Summary of validation parameters

Linearity	250-750 (μg/ml); *r* = 0.9998
Range (μg/ml)	
Linear range	250-750
Working range	21.831-750
Target range	400, 500 and 600
Target concentration	500
Precision (%RSD)	
Repeatability	0.256
Intraday	0.642
Interday	0.712
Accuracy (%recovery)	99.36
LOD (μg/ml)	7.20445
LOQ (μg/ml)	21.8317
